# German Bowel Cancer Center: An Attempt to Improve Treatment Quality

**DOI:** 10.1155/2015/456476

**Published:** 2015-05-03

**Authors:** Olof Jannasch, Andrej Udelnow, Stefanie Wolff, Hans Lippert, Pawel Mroczkowski

**Affiliations:** ^1^Department of General, Abdominal and Vascular Surgery, University Hospital, Magdeburg, Germany; ^2^Institute of Quality Assurance in Operative Medicine Ltd., Otto-von-Guericke University, Magdeburg, Germany

## Abstract

*Background*. Colorectal cancer remains the second most common cause of death from malignancies, but treatment results show high diversity. Certified bowel cancer centres (BCC) are the basis of a German project for improvement of treatment. The aim of this study was to analyze if certification would enhance short-term outcome in rectal cancer surgery. *Material and Methods*. This quality assurance study included 8197 patients with rectal cancer treated between 1 January 2008 and 31 December 2010. We compared cohorts treated in certified and noncertified hospitals regarding preoperative variables and perioperative outcomes. Outcomes were verified by matched-pair analysis. *Results*. Patients of noncertified hospitals had higher ASA-scores, higher prevalence of risk factors, more distant metastases, lower tumour localization, lower frequency of pelvic MRI, and higher frequencies of missing values and undetermined TNM classifications (significant differences only). Outcome analysis revealed more general complications in certified hospitals (20.3% versus 17.4%, *p* = 0.03). Both cohorts did not differ significantly in percentage of R0-resections, intraoperative complications, anastomotic leakage, in-hospital death, and abdominal wall dehiscence. *Conclusions*. The concept of BCC is a step towards improving the structural and procedural quality. This is a good basis for improving outcome quality but cannot replace it. For a primary surgical disease like rectal cancer a specific, surgery-targeted program is still needed.

## 1. Introduction

Colorectal cancer (CRC) remains the second most common reason of death within malignancies, but the results of treatment show high diversity [1, 2]. At the beginning of the millennium the German Cancer Society mandated the OnkoZert Institute (Neu-Ulm, Bavaria, Germany) to perform voluntary certifications of high-volume hospitals. The requirements for certification included compliance with current guidelines, detailed documentation, and an interdisciplinary approach [3, 4]. Following the year 2013 certification guidelines, about 197 qualitative and quantitative requirements have to be fulfilled by a hospital before achieving certification as a Bowel Cancer Center (BCC). The most important surgical issues concerning perioperative outcome are listed below:

1. Necessity for documented cooperation between departments of visceral surgery, gastroenterology, radiotherapy, haematology/oncology, pathology, radiology, psychotherapy, social workers, physiotherapy, genetic counselling, anesthesiology, and patient organizations.
2. At least weekly multidisciplinary tumor case conferences with pretherapeutic presentation of all colorectal cancer patients treated in any of the cooperating units.
3. Periodical morbidity and mortality conferences (biannually).
4. Pretherapeutic diagnostic standards: histological specimen of tumor, abdominal ultrasound, chest X-ray, carcinoembryonal antigen (CEA), and complete colonoscopy.
5. Histological confirmation of malignancy in 100% of patients prior to surgery.
6. Personal and volume requirements: two named qualified surgeons responsible for CRC surgery (board certification visceral surgery required); 30 colon cancer operations/year and 20 rectal cancer operations/year performed within the department; 15 colon cancer operations/year and 10 rectal cancer operations/year by each responsible surgeon.
7. Requirements for department of pathology: frozen section, definite documentation of localization, type (WHO classification), invasion (pT), regional lymph nodes (pN), number of examined lymph nodes, number of infiltrated lymph nodes, grading, residual disease (R), resection margin, lymphatic/blood vessel invasion, Mercury classification, and tumor regression after neoadjuvant treatment.

Other requirements concern institutional cooperation with radiology, endoscopy, radiotherapy, oncology, and other departments.

Two different understandings of “treatment quality” should be mentioned:

- Following OnkoZert, it is mostly characterized by structural and procedural prerequisites.
- By intuitive and established understanding, treatment quality reflects mostly outcome quality, different postoperative morbidities (e.g., the rate of serious adverse events, such as anastomotic leakage), and in-hospital death of various providers, given that the patient and illness side characteristics would not differ between these hospitals.

The evaluation of the latter, however, remains all but trivial from the methodological point of view and requires extensive auditing. This might possibly be the cause for the still lacking evidence that certified hospitals perform better than noncertified hospitals regarding treatment quality of CRC surgery. Nevertheless the number of certified hospitals is consistently rising.

We analyzed the short-term performance of certified and noncertified hospitals in rectal cancer patients registered in the international multicenter quality assurance study “colon/rectum cancer (primary tumor),” prospectively collecting treatment data about patients with colorectal cancer [5]. The study was conducted by the Otto-von-Guericke University of Magdeburg, Germany. The aim of the present investigation was to evaluate whether the certification itself would enhance the short-term outcome compared to noncertified hospitals.

## 2. Materials and Methods

The null hypothesis was that there is a better performance in certified hospitals.

All patients with rectal cancer treated between 1 January 2008 and 31 December 2010 (date of admission) were included. Exclusion criteria were colon cancer and/or treatment outside of Germany. The cohort was split according to the certification-status of the hospital performing the treatment. The patient was considered to be treated in a certified hospital when the admission date was later than the certification date [3].

In the first step, we compared cohorts treated in certified and noncertified hospitals regarding median age, ASA-classification [6], presence of at least one patient related risk factor, localization and stage of the tumor, and preoperative imaging. The stage of the tumor was described in the pathological examination of the operative specimen. Risk factors were defined according to the estimation prior to surgery and categorized into none, cardiac risk, respiratory risk, renal risk, hepatogenic risk, nicotine abuse, alcohol abuse, diabetes mellitus, varicosis, and other. Furthermore, we compared the following perioperative outcome parameters:

- At least one intraoperative complication defined as perforation of the tumor, surgical bleeding, and injury of ureter, urethra, urinary bladder, spleen, bowel, internal genital organs, and other.
- At least one general postoperative complication defined as pulmonary embolism confirmed in CT scan, respiratory complication (pleural effusion, atelectasis, pneumonia confirmed in ultrasound, chest X-ray, or CT scan), urinary tract infection (confirmed by positive microbiological sample), fever (>38°C, >2 days), cardiac complication (newly developed arrhythmia, heart failure, and pericardial effusion), thrombosis (confirmed in duplex sonography), renal complication (kidney failure), and other; anastomotic leakage requiring surgery; abdominal wall dehiscence; and in-hospital death.

Also, the quality of TME (total mesorectal excision) according to the Mercury classification [7] and R0-resections rates were compared. The peri- and postoperative results were verified by matched-pair analysis.

The statistical calculations were performed using the R software [8]. Comparisons of groups were performed with scalar variables using the *t*-test in cases of normal distribution and otherwise using the Mann-Whitney *U* test (MW*U* test). Categorical variables were tested with the Chi square test and nominal variables with Fisher's exact test. A logistic multiple regression analysis on in-hospital death, which differed between the two groups, was done using the glm function of the MASS-package [9]. The initial maximal model included all of the pretreatment variables and was stepwise reduced by eliminating the term with the highest *p* value until all terms were significant (stepwise backward algorithm). The significant terms, as well as those pretreatment variables (including pathologic TNM classification) which differed between the groups, were then balanced by matched-pairs analysis using the “matchit” function of the “MatchIt” package [10]. Differences were considered significant if *p* < 0.05.

The study was conducted according to the Declaration of Helsinki and in adherence with good clinical practice guidelines. The participation in the study was voluntary, the data collection confidential, and the data analysis anonymous. It was an observational study, without any influence on the methods or the course of treatment; therefore, as confirmed by the Ethics Committee of the University of Magdeburg, approval was not required. All patients have given their informed consent in writing for data collection and evaluation.

## 3. Results

8197 patients fulfilled the inclusion criteria.  Table 1 shows the distribution of the hospitals and patients into each of the two cohorts. The numbers and percentages of certified hospitals increased continuously during the study: in 2010 about 40% of all rectal cancer patients were treated in certified hospitals.

The cohorts treated in both types of hospitals were not equal. Patients of noncertified hospitals had higher ASA-scores, a higher prevalence of risk factors, more distant metastases, lower localization of tumors, a lower frequency of pelvic MRI and higher frequencies of missing values, and undetermined TNM classifications (significant differences only). Details are given in Table 2.

Therefore, it was necessary to match the patients of both cohorts by balancing the most important explanatory variables. We performed logistic multiple regression analysis and revealed that gender, age, body weight, comorbidities, ASA-classification, liver metastases, and neoadjuvant treatment influenced the postoperative death risk in rectal cancer patients (data not shown).

We used the “matchit” function of the respective R package via “nearest neighbor” gradients to balance these variables. The explanatory variables of matched rectal cancer subcohorts (in both subcohorts *N* = 1807) are shown in the right part of Table 3. Of the balanced variables, none differed significantly between the certified and noncertified hospitals. However, pelvic MRI was performed significantly more often in the certified group. Furthermore, the distributions of T classifications showed more cases with T3 in the noncertified hospitals. In the outcome analysis, certified hospitals revealed significantly more general complications (20.3% versus 17.4%, *p* = 0.03). Both cohorts did not differ significantly in the percentage of R0-resections, intraoperative complications, anastomotic leakage, in-hospital mortality, and abdominal wall dehiscence.

## 4. Discussion 

The German Cancer Society has initiated the formation of specialized high-volume centers by certifying quality of care aiming to achieve better treatment results [11]. For breast cancer care, the achievement of quality indicators (QIs) in breast centers was analyzed from 2003 until 2007 and an improvement in QI achievement was reported [12]. The differences in pretreatment patient characteristics as well as the comparison with noncertified hospitals were not taken into consideration. Therefore, the aim of our investigations was to compare the outcome of rectal cancer treatment in certified and noncertified hospitals by parameters indicating the quality of care.

The analysis of both cohorts revealed gradual but significant differences in ASA-classification, comorbidities, and tumor stage. It turned out that noncertified centers treated patients with slightly higher perioperative risks compared to certified centers. This had to be taken into consideration for comparisons of both cohorts. It seems that the diagnostic algorithms of certified hospitals adhered more tightly to the current guidelines than those of noncertified hospitals (rate of preoperative pelvic MRI and complete TNM classification). If these parameters would be considered as QIs of CRC care, certified hospitals would have clearly performed better. Nevertheless, the surgical outcome is traditionally measured by morbidity, mortality, and healing. In order to compare the cohorts by these parameters we had to equalize both cohorts concerning the main risk factors by matched-pairs analysis. Table 3 reflects that noncertified hospitals had fewer general complications.

Although conclusions from observational studies should always be drawn with caution, we can state that an improvement of surgical quality is not visible in our comparison of certified versus noncertified hospitals. There are several possible sources of bias. First, the certification itself reflects adherence to a given list of parameters [13], mostly reflecting the structure and process quality. The most sensitive direct parameter of surgical quality recorded by the BCC is quality of the specimen in rectal cancer. It is set at a surprisingly low level [14], requiring only 70% of specimens classified as good or moderate according to the Mercury classification [15]. This implies that, on average, in every third patient a poor quality of the specimen is accepted and that a hospital regarded as fulfilling the certification criteria does not have to produce any single good TME specimens. This seems inconsistent in the context of the proven relationship between the quality of the specimen and the risk of a local relapse [7, 16]. Furthermore, there is no declaration about a standardized pathological protocol used for the evaluation of a specimen.

The certification criteria impose the minimal annual volume of 20 rectal cancer and 30 colon cancer operations. A BCC must employ a minimum of 2 qualified surgeons (visceral surgeons according to the German board certification), each of whom must perform a minimum of 10 rectal cancer and 15 colon cancer operations annually. Newly appointed surgeons at a certified hospital must have performed a total of 10 rectal cancer and 15 colon cancer operations within the last 3 years [13]. Hospital and surgeon volumes are discussed as an influential factor of surgical quality; however, there are no unified definitions of volume groups. The recently published Cochrane analysis [17] confirms a general trend to better results in higher hospital and surgical volume as well as with surgeon specialization. However, some of the analyzed studies defined high-volume hospital as more than 20 operations annually, while other studies set this limit at more than 150 operations. Similar large differences exist for surgeon volumes, and there is also no unified definition for a specialized colorectal surgeon. The Cochrane group observed a trend that the effect of hospital volume is present mostly in US-based analyses, but not in European studies. This corresponds with the previous findings of our group [18] and with the latest data from the Swedish registry [19], suggesting that participation in a quality assurance program diminishes the volume effect. This could also be a partial explanation of the present findings, as the certified hospitals are not compared to a randomly assessed group of hospitals, but both cohorts do participate in the quality assurance program [5].

## 5. Conclusions

The questions remains: Is there any benefit of certification in this version of the BCC? The significant differences in the use of pretherapeutic MRI and in the percentage of missing values in pathological classifications demonstrate that the certification leads to a better concentration of treatment-related issues. This at least is a benefit for the patient. The German concept of BCC is a step towards improving the structural and procedural quality. Both of these aspects are a good basis for improving the outcome quality but cannot replace it. For a primary surgical disease like rectal cancer, a specific, surgery-targeted program is still needed. Possibly participation in a quality assurance program is a first step in improving outcome and this should be also the primary effect of any certification.

## Figures and Tables

**Table 1:** Structure and distribution of patients and hospitals.

Year	Certified		Noncertified	
	*n*	%	*n*	%
Hospitals				
2008	30	18.5	132	81.5
2009	40	27.6	105	72.4
2010	43	31.6	93	68.7

Patients				
2008	582	19.3	2422	80.7
2009	804	30.3	1853	69.7
2010	1025	40.4	1511	59.6

**Table 2:** Tumour and patient related factors.

	Certified	Noncertified	*p*
Age (mean in years)	68.3	68.7	0.17^∗^
ASA I (%)	8.1	6.9	0.069^∗∗^
ASA II (%)	53.0	49.1	0.0025^∗∗^
ASA III (%)	36.2	40.4	0.0002^∗∗^
ASA IV (%)	2.2	2.7	0.29^∗∗^
Min. 1 risk factor (%)	76.7	79.9	0.002^∗∗^
CT (%)	81.9	84.0	0.025^∗∗^
Pelvic MRI (%)	38.9	29.3	<0.001^∗∗^
Neoadjuvant treatment (%)	41.0	36.5	<0.001^∗∗^

Tumour localization (cm from anal verge)			
<4 cm (%)	12.9	15.2	0.008^∗∗^
4–7.9 cm (%)	29.7	28.8	0.42^∗∗^
8–11.9 cm (%)	30.4	29.1	0.25^∗∗^
12–16 cm (%)	26.9	26.8	0.91^∗∗^

TNM classification			
pTis	4.65	4.35	0.55^∗∗^
pT1	13.7	12.1	0.06^∗∗^
pT2	24.8	24.3	0.69^∗∗^
pT3	44.7	46.4	0.14^∗∗^
pT4	9.3	9.7	0.59^∗∗^
Missing pT	1.6	2.3	0.034^∗∗^
pN0	57.4	56.0	0.228^∗∗^
pN1	20.8	20.9	0.90^∗∗^
pN2	16.7	16.9	0.87^∗∗^
pNX	3.0	4.0	0.03^∗∗^
Missing pN	2.0	2.2	0.73^∗∗^

^∗^Mann-Whitney*U* test; ^∗∗^Fisher's exact test.

**Table 3:** Short-term outcome morbidity, R0-state, and in-hospital death.

	Before matching			After matching		
	Certified	Noncertified	*p*	Certified	Noncertified	*p*
At least 1 intraoperative complication (%)	4.6	5.3	0.19	2.7	2.7	0.68
General complications (%)	19.3	18.6	0.457	20.3	17.4	0.03
Abdominal wall dehiscence (%)	1.8	1.9	0.788	1.7	1.6	0.90
Anastomotic leakage (%)	4.4	4.3	0.766	4.4	4.5	0.94
R0 resection (%)	83.5	80.2	0.00057	85.3	85.7	0.78
In-hospital death (%)	2.6	3.5	0.05528	2.8	2.7	1

*p* for Fisher's exact test.
